# Infusion of Non-HLA-Matched Off-the-Shelf *Ex Vivo* Expanded Cord Blood Progenitors in Patients Undergoing Cord Blood Transplantation: Result of a Phase II Clinical Trial

**DOI:** 10.3389/fcell.2022.835793

**Published:** 2022-04-04

**Authors:** F. Milano, L.A. Thur, J. Blake, C. Delaney

**Affiliations:** ^1^ Clinical Research Division, Fred Hutchinson Cancer Research Center, Seattle, WA, United States; ^2^ Department of Medicine, University of Washington School of Medicine, Seattle, WA, United States; ^3^ Deverra Therapeutics, Seattle, WA, United States; ^4^ Department of Pediatrics, University of Washington School of Medicine, Seattle, WA, United States

**Keywords:** cord blood, cellular therapy, hematopoietic recovery, expansion stem cells, allogeneic transplantation

## Abstract

Recipients of myeloablative cord blood transplants (CBT) are known to experience delayed hematopoietic recovery and an increased risk of transplant related mortality (TRM). We developed methods for *ex vivo* expansion and cryopreservation of CB stem and progenitor cells. 15 patients with hematologic malignancies were enrolled in this single center phase II trial between September 2010 and August 2012 to assess the safety of infusing a non-HLA-matched expanded CB product to bolster a conventional CBT. On the day of transplant, an infusion of the expanded CB product followed the primary graft (1 or 2 unmanipulated CB units). All patients engrafted. Median time to neutrophil and platelet recovery was 19 and 35 days, respectively. Early myelomonocytic recovery was almost entirely due to cells arising from the non-HLA-matched expansion product and were no longer detected at day 14 in all but 2 patients. The probability of 3-years disease free survival was 86%. No TRM was observed throughout the study period, and only 2 patients relapsed. All patients presented with grade II acute graft-versus-host disease (aGVHD) at a median time of 32 days, with no grade III-IV aGVHD observed. At 2 years only 2 patients remain on immunosuppressive therapy for mild chronic GVHD. This phase II safety study demonstrate that infusion of an off-the-shelf non-HLA-matched expanded CB product in addition to a conventional CB graft was safe and led to sustained myeloid recovery. Based on these encouraging results, a prospective multicenter randomized trial utilizing this product has been conducted and results will be soon released. ClinicalTrials.gov Identifier: NCT01175785.

## Introduction

Allogeneic hematopoietic cell transplantation (HCT) remains the only known curative approach for patients with high-risk leukemia. However, most patients who require HCT lack a suitable HLA-matched sibling and identification of an unrelated donor remains a challenge for racial and ethnic minority patients (Gragert et al., 2014). For these patients, banked cord blood (CB) donors represent a critical source of stem cells for both in pediatric and adult patients (Kurtzberg et al., 1996; Laughlin et al., 2001; Laughlin et al., 2004; Rocha et al., 2004). Important advantages of CB transplantation (CBT), such as rapid donor identification and availability are often counterbalanced negatively by the low stem cell dose present in the CB graft leading to delayed hematopoietic recovery, particularly neutrophil recovery (ANC) (Dahlberg and Milano 2017). This leaves patients susceptible to infection in the early period post-transplant (Ruggeri et al., 2011). To better establish sufficient stem cell numbers for reliable donor engraftment, it is now common for two partially HLA-matched CB units to be infused to achieve an adequate hematopoietic stem and progenitor cell dose (HSPC) (Barker et al., 2005). However, the time to donor engraftment is still delayed despite the modest two-fold increase provided by the second CB unit, and the risk of early transplant related mortality (TRM) is increased when compared to conventional donor sources (Brunstein et al., 2010).

The stem cell biology field is rapidly advancing, and many have explored methods of manipulating CB HSPC to enhance the potential for engraftment and immune reconstitution (Frassoni et al., 2008; Boitano et al., 2010; Cutler et al., 2011; de Lima et al., 2012; Horowitz et al., 2013; Cohen et al., 2020). This avenue of research has principally focused on stimulating progenitor cell self-renewal and blocking differentiation into more mature cell types with the goal of HSPC expansion. This work resulted in the development of a pre-clinical Notch-mediated expansion system for hematopoietic progenitors using the Notch ligand Delta (Delaney et al., 2010). Delaney et al. utilized Notch ligand Delta expansion in a first-in-human phase I CBT trial using patient-specific *ex vivo* expanded CB progenitors and an unmanipulated CB unit following myeloablative conditioning (Delaney et al., 2010). The outcomes from this phase 1 trial demonstrated safety of this approach as well as a significant reduction in time to neutrophil recovery (Delaney et al., 2010). These promising results relied on real-time and patient-specific expansion of a CB unit, which is not feasible on a large-scale. Therefore, for this clinical trial we utilized an expanded, cryopreserved product that is infused without HLA-matching as an adjuvant source of rapidly repopulating hematopoietic progenitors when infused following one or two unmanipulated CB units. Here we report the final results of a phase-II pilot study exploring the safety and efficacy of the infusion of off-the shelf *ex-vivo* expanded cryopreserved CB HSPC as part of single and double CBT.

## Methods

### Study Design

We conducted a prospective open-label single arm study to assess the feasibility, safety, and preliminary efficacy of a non-HLA-matched, previously *ex vivo* expanded and cryopreserved CB progenitor cell product infusion in patients following a single or double CBT. The primary endpoints were to examine the safety and toxicity when *ex vivo* expanded cord blood cells were infused as an off-the-shelf, non-HLA-matched product. We also sought to determine the *in vivo* persistence of the *ex vivo* expanded CB product along with its contribution to engraftment by weekly determination of donor chimerism in the peripheral blood.

### Patients

Patients who were 6 months to ≤45 years of age and had high-risk acute lymphocytic leukemia (ALL), acute myeloid leukemia (AML), chronic myeloid leukemia (CML), or myelodysplastic syndrome (MDS) were eligible. Patients were enrolled only if they did not have a matched related or unrelated donor. This study was funded by the National Heart, Lung, and Blood Institute (NHLBI) and had the priority over conventional CBT. Patients were required to have an adequate performance status, and organ function. Those eligible must also have access to one or two CB units matched at four of more HLA loci by intermediate-resolution for HLA class I alleles (A and B) or by high-resolution typing for the HLA class II DRB1 allele. Single unit CBT was permitted for 6/6 units with total nucleated cell count (TNC) ≥ 2.5 × 10^7^/ kg, 5/6 and 4/6 units with TNC ≥ 4.0 × 10^7^/ kg. If these thresholds were not met, double CBT was performed, with each unit required to have a TNC ≥1.5 × 10^7^ /kg. All subjects provided written informed consent to participate in the study, which was approved by Fred Hutchinson Cancer Research Center’s Institutional Review Board (IRB). Details of the inclusion and exclusion criteria are provided in http://clinicaltrials.gov (Identifier: NCT01175785).

### *Ex-Vivo* Expanded Progenitor Cell Products: Cell Processing and Manufacturing

Manufacturing of the expanded progenitor cell product (EPC) from a single donor CB, processing and CD34 cell selection, culture initiation, *ex vivo* expansion, final cell product harvesting and cryopreservation were conducted as per FDA-approved IND 14184 (Sponsor C Delaney). Briefly, human CB samples were obtained from normal full-term deliveries with IRB approval and donor eligibility determined as per 21CFR1271 by the Puget Sound Blood Center Cord Blood Bank. The CB units were red cell depleted and underwent clinical grade selection of CD34 ^+^ cells using the Miltenyi CliniMACS per the manufacturer’s instructions. The negative fraction was discarded.

Cultures were initiated with the purified CD34 ^+^ cells and cultured for 14–16 days in non-tissue culture treated tissue culture flasks (Nunc, Thermo Fisher Scientific, Pittsburg, United States). Culture vessels were pre-coated with clinical grade Notch ligand (Delta1^ext−IgG^, prepared in the Fred Hutchinson’s Biologics Production Facility, DMF BB-MF 12366) (Delaney et al., 2005) and fibronectin fragment CH-296 (Takara Shuzo Co. Ltd., Otsu, Japan) and then washed with PBS. Cells were cultured in serum-free medium (Stemspan SFEM, Stemcell Technologies, Vancouver, BC, Canada) with clinical grade recombinant human IL-3, IL-6, Thrombopoietin (TPO), Flt-3 Ligand and Stem Cell Factor (SCF) (CellGenix Freiburg, Germany). Cells were split into new culture vessels to maintain a cell density of < 1 × 10^6^ total cells per milliliter of media.

On day 14–16 of culture, the total volume of cells was harvested, and final release testing performed, including final cell counts and calculation of CD34 and TNC fold expansion, immunophenotyping, bacterial and fungal sterility and endotoxin as per FDA approved IND. The product was then cryopreserved in a controlled rate freezer and banked for future use.

### Transplant Procedures: Conditioning Regimen, Graft-Versus-Host Disease Prophylaxis and Kinetics of Engraftment

**FIGURE 1:**
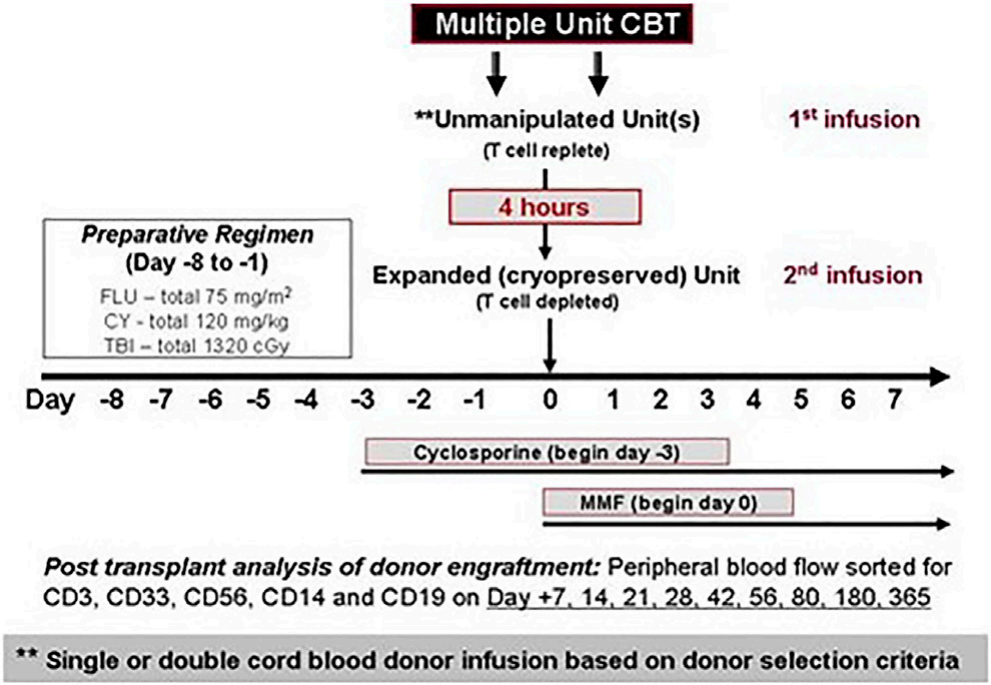
Treatment plan.

All patients received myeloablative conditioning with cyclophosphamide 120 mg/kg and total body irradiation 1,320 cGy with the addition of fludarabine 75 mg/m^2^. On the day of transplant (Day 0), the patient underwent infusion of one or two CB units as the primary graft source followed 4 h later by the infusion of the off-the shelf expanded progenitors’ cells graft (EPC) graft (Figure 1). Prophylaxis for GVHD consisted of cyclosporine beginning on day -3 and continuing for a minimum of 180 days, and mycophenolate mofetil (MMF) beginning on day 0 and administered until at least day 45 or potentially longer in the presence of active GVHD.

Time to neutrophil engraftment was defined as the first of two consecutive days with an absolute neutrophil count (ANC) of 0.5 × 10^9^ per liter or higher, and time to platelet engraftment as the first of 7 consecutive days with an unsupported platelet count of 20 × 10^9^ per liter or higher. Post-transplant granulocyte-colony stimulating factor (G-CSF) was given until the absolute neutrophil (ANC) recovery to >2.5 × 10^9^/ L was stable for 3 days, and then reduced to as needed while maintaining an ANC >1.0 × 10^9^/ L.

Chimerism testing was performed on peripheral blood samples that were sorted by FACS into CD3^+^, CD33^+^, CD14^+^, CD56^+^ and CD19 ^+^ cell fractions on days +7, +14, +21, +28, +42, +56, +80, +180 and 1 year using a DNA-based assay for short tandem repeat (STR) loci. Day +28, +42 and +80 were done only if previous chimerism did not exhibit a minimum of 95% engraftment from a single cord blood donor.

### Statistical Analysis

Probability of disease-free survival (DFS) was calculated using the method of Kaplan and Meier (Kaplan and Meier 1958). Cumulative incidence of relapse, non-relapse mortality (NRM), and acute GVHD were summarized using cumulative incidence estimates (Lin 1997; Fine and Gray 1999), with relapse regarded as a competing risk for NRM, NRM a competing risk for relapse, and death without failure for each of the other endpoints regarded as a competing risk for each, respectively. All statistical analyses were performed using STATA 16.0 (StataCorp, College Station, TX).

## Results

### Patients

Table 1 shows the characteristics of the 15 patients who, from September 2010 to August 2012, received a single or double CBT along with EPC. The median follow-up of surviving patients was 8.5 years (range, 5–11 years). Patients were 5–45 (median 21) years old and weighed 23–89 (median 59) kg. Diseases included AML (*n* = 6), ALL (*n* = 8), MDS (*n* = 1). Six patients (40%) had measurable residual disease (MRD) at the time of transplant, defined as presence of disease assessed by ten-color multiparameter flow cytometry on bone marrow aspirates obtained as a routine baseline before HCT. All but 4 patients (27%) received two CB units to achieve the required cell dose.

**TABLE 1:** Patient and unit characteristics in recipients of *ex-vivo* expanded cells.

Characteristic	EPC (*n* = 15)
Female, n (%)	7 (46)
Age, y, median (range)	21 (5–45)
Weight, kg, median (range)	59 (23–89)
Diagnosis, n (%)	
ALL	8 (53)
AML	6 (40)
MDS/CML	1 (7)
Other	0
CMV seropositive, n (%)	11 (73)
Race, n (%)	6 (40)
Caucasian	9 (60)
Non-Caucasian	
Disease Risk, n (%)	-
Low	11 (74)
Intermediate	3 (20)
High	1 (6)
Very high	
MRD+, n (%)	6 (40)
Follow-up, y, median (range)	5.7 (4.4–6.2)
Number of unmanipulated donors, n (%)	
1	4 (27)
2	11 (73)
HLA match unmanipulated donors, n (%)^a^	
4/6	9 (60)
5/6	6 (40)
6/6	-
Infused Cell Doses (pre-freeze), median (range)^b^	
Total Unmanipulated TNC/kg x 10^7^	6.1 (4.3–17.1)
Total Unmanipulated CD34/kg x 10^6^	0.26 (0.08–0.98)
Expanded Product TNC/kg x 10^7^	5.8 (2.2–10.9)
Expanded Product CD34/kg x 10^6^	5.2 (3.1–11.6)

ALL, acute lymphoblastic leukemia; AML, acute myeloid leukemia; MDS/CML, Myelodysplastic Syndrome/Chronic Myeloid Leukemia; MRD, measurable residual Disease; HLA.

a HLA, matching reflects the lowest HLA-match of the unmanipulated unit.

b Pre-freeze median TNC, and CD34^+^ of all units.

### ECP Expansion and Infusion

Fifteen products were generated from twelve manufacturing runs to support product for this trial. At the time of harvest of the expanded cell product, there was an average fold expansion of CD34 ^+^ cells of 141 (±16, s. e.m., range 68–240) and an average fold expansion of total cell numbers of 1099 (±90, s.e.m., range 552–1,434). The composition of the non-HLA matched expanded cell graft was composed mainly of CD34 ^+^ HSPCs (∼30–35%) and myeloid progenitors. The exact immunophenotypic composition is proprietary. Of note, there were no mature T cells infused with the expanded graft as the product consisted of the total progeny generated after culture of purified CD34 ^+^ HSPC isolated from a single CB unit. The negative fraction, including the T cells, were not retained and no T cells were generated or maintained in the 14-days culture period. The median pre-freeze TNC and CD34 ^+^ cell dose derived from the EPC was 5.8 × 10^7^/ kg (range 2.2–10.9) and 0.26 × 10^6^/ kg (range 3.1–11.6), respectively. The median pre-freeze TNC and CD34 ^+^ cell dose derived from the unmanipulated graft was 6.1 × 10^7^/ kg (range 4.3–17.1) and 0.26 × 10^6^/ kg (range 0.08–0.98), respectively.

### Hematopoietic Recovery and Chimerism

**FIGURE 2:**
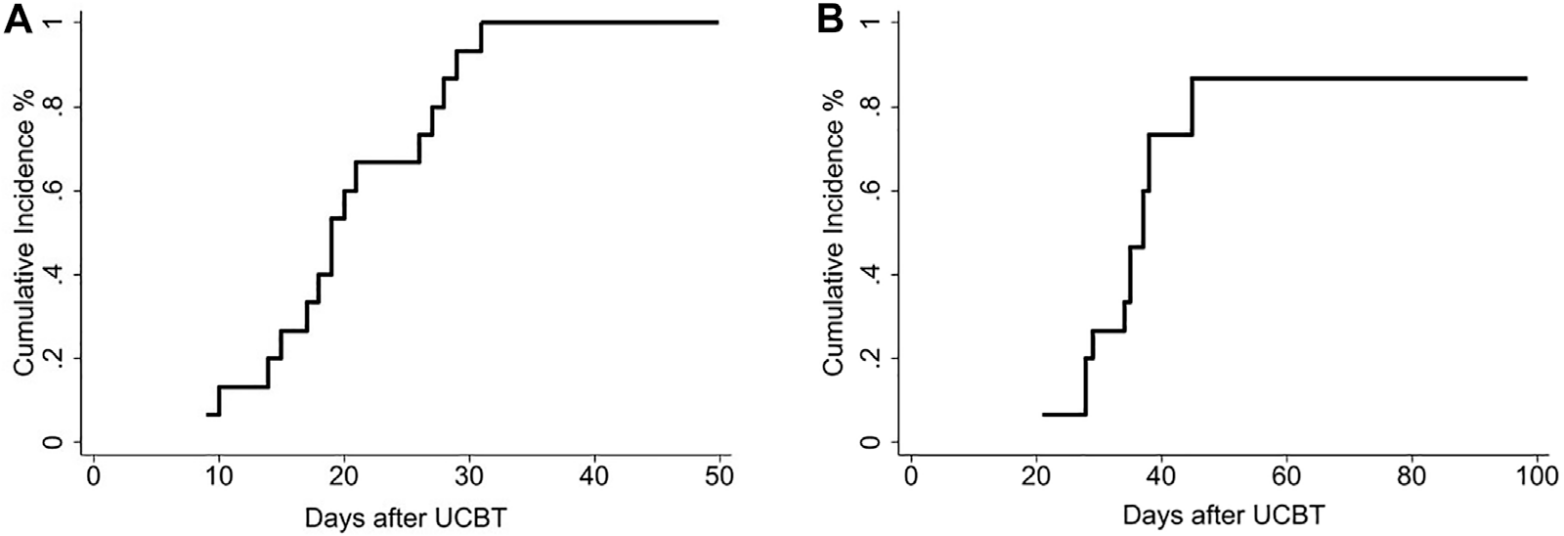
A total of 15 patients who received 1 or 2 unmanipulated CB units along with a randomly selected non-HLA-matched expanded CB unit. Figure 2A shows the cumulative incidence of neutrophil recovery. Figure 2B shows the cumulative incidence of platelet recovery.

The median time to neutrophil recovery was 19 days (range 9–31) in the recipients of EPC. The median time to platelet engraftment was 35 days (range 21–86) in the recipients of EPC (Table 2). Furthermore, day 100 cumulative incidence of neutrophil and platelet engraftment was 100 and 93%, respectively (Figures 2A, B).

**TABLE 2:** Engraftment in recipients of *ex-vivo* expanded cells.

Neutrophil Engraftment	*n* = 15
Time to Engraftment, d, Median (range)	19 (9–31)
Cumulative Incidence, % (95% CI) By 100 days	100 (61–100)
Platelet Engraftment	*n* = 14
Time to Engraftment, d, Median (range)	35 (21–86)
Cumulative Incidence, % (95% CI)	80 (50–93)
By 60 days	93 (61–99)
By 100 days	

**FIGURE 3:**
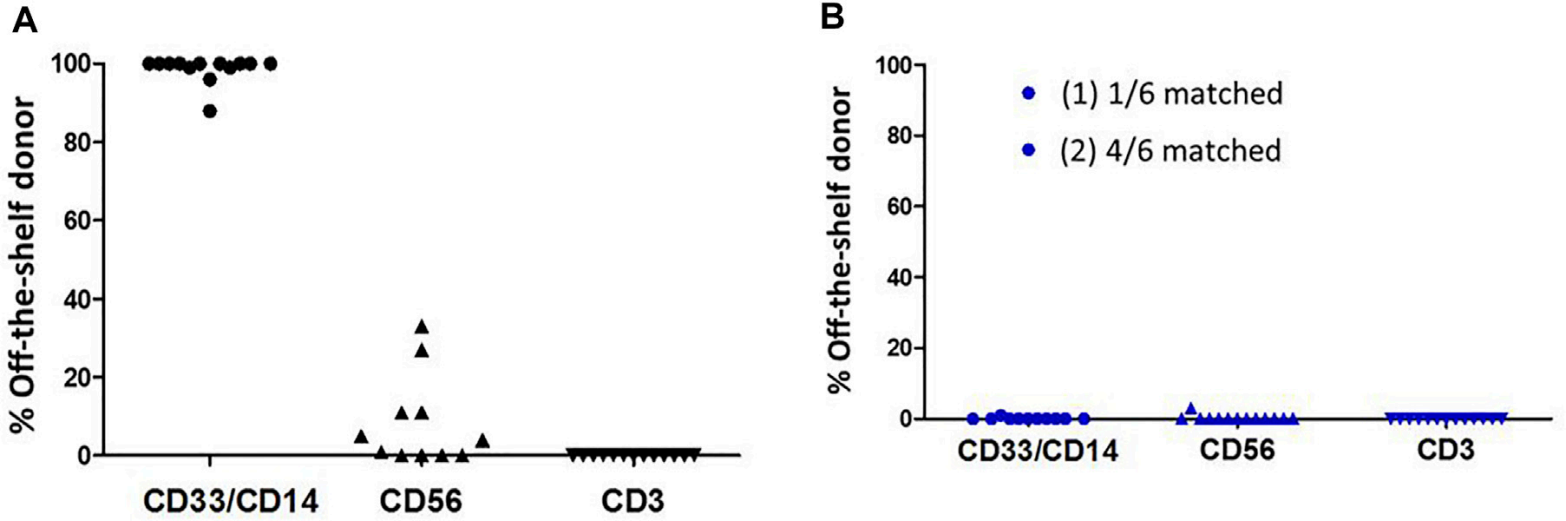
Persistence in peripheral blood of cells derived from the expanded cell graft at day 7 (Figure 3A) and at day 14 (Figure 3B).

Sorted peripheral blood chimerisms performed weekly in the first month post-transplant beginning on day 7, uniformly demonstrated that early myelomonocytic engraftment (day 7, CD33 and CD14 cell fractions) was derived almost entirely from the EPC product. No contribution to CD3 from the EPC product was seen, as expected, and contribution to CD56 derived from the EPC product was variable. Early (day 7) myelomonocytic (CD33 and CD14) recovery was almost entirely (98–100%) due to cells arising from the expansion product (Figure 3A). Cells derived from the expansion product were no longer detected at day 14 in all but 2 patients (Figure 3B).

### Transplant Outcomes

**FIGURE 4:**
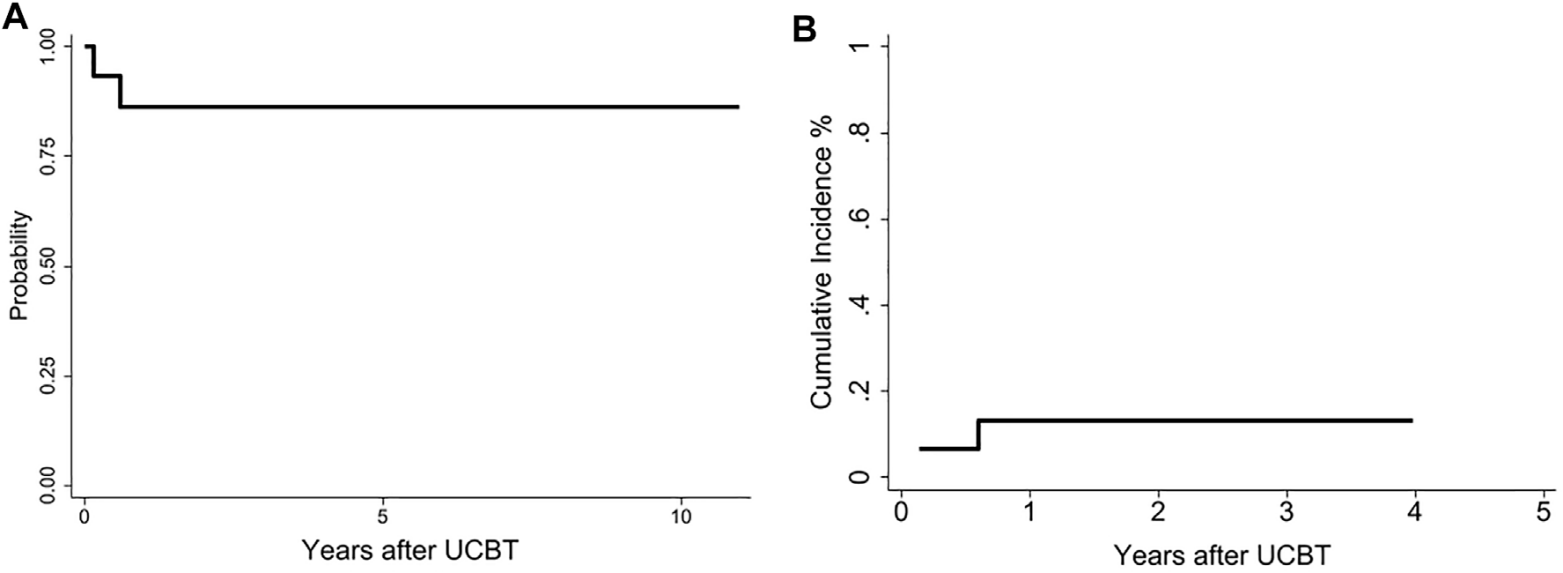
Disease free survival at 10 years: recipients *ex-vivo* expanded cells 86% (95% CI: 56–96) (Figure 4A). Cumulative incidence relapse (Figure 4B).

The probability of 3-years DFS was 86% (95% CI: 57–97) (Figure 4A) and there was no NRM was observed during the study period. Two patients relapsed at days 53 and 219 posttransplant and subsequently died after further therapy. The patient who relapsed at day 53 had circulating blasts at the start of conditioning (Figure 4B).

All patients were diagnosed with maximum grade II acute GVHD at a median time of 32 days (14–86), with no grade III-IV aGVHD observed. The most commonly affected organ was the skin in the group receiving the EPC (*n* = 12). Eight (53%) patients were treated for pre-engraftment syndrome at a median time of 6 days (range 4–9) and five (33%) patients had GVHD after day 100. After day 100, of the 13 patients evaluable, 3 (23%) had late aGVHD features or an overlap syndrome, while none were diagnosed with features of classical chronic GVHD. At 2 years, nine of 13 evaluable patients (70%) were off immunosuppression.

## Discussion

This is the first report on the use of an off-the-shelf non-HLA-matched expanded CB product as part of single and double CBT. In our study, the combination of the EPC with unmanipulated CB units was associated with rapid ANC and platelet engraftment when compared to our historical control group and to results reported by other groups using only unmanipulated CB units (Barker et al., 2005).

Similar to our initial expansion trial using a patient-specific Notch-expanded graft that was infused fresh (Delaney et al., 2010), early myeloid and monocytic engraftment was derived from the expanded cell graft as demonstrated by weekly peripheral blood cell sorted chimerism analysis. However, in the case of the off-the-shelf, non-HLA-matched EPC cells derived from the expansion product no longer detected at day 14 in the peripheral blood in all but 2 cases. In addition, all patients receiving a double unit CBT were single donor dominant by day 21. Although single donor dominance is expected after conventional unmanipulated dCBT, the kinetics of single donor dominance was enhanced in patients receiving EPC in addition to the unmanipulated grafts and suggests an enhancement of the immune-mediated graft-vs-graft phenomenon described previously by our group (Gutman et al., 2010). The lack of *in vivo* persistence despite the higher CD34 cell dose is not surprising given that the EPC is an allogeneic non-HLA matched product that is also devoid of donor T cells to mitigate the risk of immune mediated rejection by the unmanipulated T cell replete CB grafts. Despite the loss of contribution to chimerism, we still observed a rapid ANC and platelet engraftment suggesting a cooperative effect of the cultured cells in promoting engraftment from the unmanipulated units. The median time to engraftment of 19 days is slightly faster than what was observed in past studies (Barker et al., 2005) and closer to what has been recently reported (Ballen et al., 2020; Milano et al., 2020). The recent improvement in time to engraftment is likely to be related to a better selection of CB units (Politikos et al., 2020).

In addition to improving ANC and platelet engraftment, no TRM was observed among the 15 patients on this study, although we did observe two deaths due to relapsed disease, thus demonstrating an excellent overall survival. The encouraging outcomes observed can be attributed to shortening the delayed time to engraftment that is often experienced by patients undergoing a CBT. However, we cannot exclude that the co-infusion of the EPC led to augmenting the graft-versus graft interactions and consequently increasing the graft-versus-leukemia effect (Milano et al., 2016). While we cannot draw definitive conclusions based on this small cohort, we are encouraged by the results with these high-risk patients (about half of them were MRD + at the time of transplant) and the long follow-up period.

This study was the first to evaluate the infusion of an off-the-shelf, non-HLA-matched progenitor cell therapy to supplement the primary CB graft with the goal of enhancing the kinetics of hematopoietic recovery and reducing the risk of early TRM. There were concerns regarding the infusion of an off-the shelf, non-HLA-matched cellular therapy, such as, the possibility of infusion toxicity, transfusion associated GVHD, alloimmunization, and immediate rejection of the expanded product. However, we observed no infusion-related toxicities, and no serious adverse events were attributed to the EPC product. As expected, the EPC contributed to the initial wave of hematopoiesis, but was generally not detected beyond 14 days. Perhaps the most surprising observation was that none of the patients included in the study experienced grade III-IV aGVHD. Rapid engraftment and equivalent transplant outcomes were also observed among the four patients receiving a single unmanipulated unit along with the EPC product. We could speculate that the use of EPC has the potential to benefit single CBT by allowing a less stringent algorithm for unit selection (utilization of smaller units) and by favoring the selection of better matched CB units. At this regard, we are currently investigating the role of ECP solely in the setting of single CBT with the goal of assessing if smaller and better matched CB units can be supported by the ECP. Furthermore, the ability to use cryopreserved, non-HLA-matched cell therapy products allows for more cost-effective manufacturing and the generation of a bank of fully released and qualified products available for immediate on-demand use.

In summary, we demonstrated that infusion of a non-HLA-matched off-the-shelf expanded CB unit is feasible and leads to enhanced and rapid engraftment in patients undergoing CBT. Based on these positive results, a prospective multicenter randomized trial comparing this approach to conventional CBT has been conducted and results will be soon released once all patients will have reach an adequate follow-up period.

## Data Availability

The raw data supporting the conclusions of this article will be made available by the authors, without undue reservation.
